# Neuromedin U Partly Mimics Thyroid-Stimulating Hormone and Triggers Wnt/β-Catenin Signalling in the Photoperiodic Response of F344 Rats

**DOI:** 10.1111/jne.12116

**Published:** 2013-11-25

**Authors:** G Helfer, A W Ross, P J Morgan

**Affiliations:** Rowett Institute of Nutrition and Health, University of AberdeenBucksburn, Aberdeen, UK

**Keywords:** Neuromedin U, photoperiod, F344 rat, hypothalamus, wnt signalling, thyroid-stimulating hormone

## Abstract

In seasonal animals, photoperiod exerts profound effects on physiology, such as growth, energy balance and reproduction, via changes in the neuroendocrine axes. A key element of the photoperiodic response is the thyroid hormone level in the hypothalamus, which is controlled via retrograde transport of thyroid-stimulating hormone (TSH) from the pars tuberalis of the pituitary. TSH regulates type II deiodinase (Dio2) expression, which transforms inactive thyroid hormone to its active form, via TSH receptors expressed in the ependymal cells of the hypothalamus. In the present study, we hypothesised that a second peptide hormone, neuromedin U (NMU), may play a role in the photoperiodic response alongside TSH because the gene for NMU is also expressed in a strongly photoperiod-dependent manner in the pars tuberalis and its receptor NMU_2_ is expressed in the ependymal layer of the third ventricle in photoperiod-sensitive F344 rats. Consistent with other studies conducted in nonseasonal mammals, we found that acute i.c.v. injections of NMU into the hypothalamus negatively regulated food intake and body weight and increased core body temperature in F344 rats. At the same time, NMU increased *Dio2* mRNA expression in the ependymal region of the hypothalamus similar to the effects of TSH. These data suggest that NMU may affect acute and photoperiodically controlled energy balance through distinct pathways. We also showed that TSH inhibits the expression of type III deiodinase (Dio3) in F344 rats, a response not mimicked by NMU. Furthermore, NMU also increased the expression of genes from the Wnt/β-catenin pathway within the ependymal layer of the third ventricle. This effect was not influenced by TSH. These data indicate that, although NMU acts with some similarities to TSH, it also has completely distinct signalling functions that do not overlap. In summary, the present study of NMU signalling reveals the potential for a new player in the control of seasonal biology.

For most organisms, survival in a seasonally changing environment requires adaptations to their physiology and behaviour. In seasonal mammals, this includes robust photoperiod-driven changes in food intake, body weight and reproduction. In the last decade, considerable progress has been made in understanding the molecular mechanisms underlying the neuroendocrine driven changes in seasonal physiology. Our knowledge of the primary effects of photoperiod on the neuroendocrine hypothalamus originates from experiments in Japanese quail, where it was shown that photoperiod regulates a novel thyroid hormone (TH) metabolic circuit 1. A critical component is thyroid-stimulating hormone (TSH), also known as thyrotrophin, derived from the pars tuberalis gland of the pituitary. TSH acts through receptors expressed in the tanycytes of the ependymal region lining the third ventricle of the hypothalamus to control type II deiodinase (Dio2) expression 1,2, thereby constituting a novel pituitary (pars tuberalis)–hypothalamus axis. Subsequently, this has been confirmed in mammals (sheep and mice), where it has been shown that melatonin produced in the pineal gland acts on the pars tuberalis to control the thyroid hormone signalling system 3,4. Dio2 catalyses the deiodination of inactive thyroxine (T4) to biologically active triiodothyronine (T3) and thus regulates thyroid hormone bioactivity and availability in the hypothalamus. In summer-like conditions (long days; LD), increased expression of Dio2 is considered to increase T3 availability within the hypothalamus. Conversely, in winter-like conditions (short days; SD), Dio2 expression decreases, whereas that of type III deiodinase (Dio3) increases. Because Dio3 de-iodinates bioactive T3 to inactive di-iodothyronine (T2), it is anticipated that this leads to a decrease in hypothalamic T3 levels in SD. Changes in Dio2 and/or Dio3 expression between long-day and short-day photoperiods have been shown in a number of seasonal species 2,4–8 but, thus far, only Dio2 has been shown to be regulated by TSH. From these data, it is now generally concluded that the TSH-driven changes in Dio2 expression, and thus increased central T3 availability, is responsible for the seasonal changes in body weight, food intake and reproduction 9.

In photoperiod-sensitive Fischer (F)344 rats, a second peptide hormone, neuromedin U (NMU), is also expressed in the pars tuberalis of the pituitary and under strong photoperiodic regulation 10. NMU shows changes in expression of a similar magnitude and temporal pattern to TSH in response to altered photoperiod 7,10 and this strongly suggests that NMU might serve a similar role to TSH in photoperiodic pituitary-hypothalamic regulation. Most physiological effects of NMU have been observed after the central administration of NMU into nonphotoperiodic rats or mice. These studies show that NMU reduces food intake and body weight and concomitantly induces body temperature, motor activity and oxygen consumption 11. The action of NMU is assumed to be mediated by the hypothalamic G-protein coupled NMU_2_ receptor (NMU_2_) 12, which is expressed in the ependymal layer of the third ventricle 13, the same hypothalamic region as the TSH receptor (TSH-R). Recently, we have shown that NMU and NMU_2_ are strongly photoperiodically regulated in the hypothalamus of F344 rats, with the highest levels in LD conditions 10. However, whether NMU plays a role in the neuroendocrine control of seasonal physiology remains unclear because, against expectations, the highest levels of NMU expression in the pars tuberalis are associated with the highest levels of food intake under LD.

In the present study, we hypothesised that NMU might play a role in the photoperiodic response similar to TSH. We investigated the effect of i.c.v. injections of NMU into the third ventricle of the hypothalamus of F344 rats held in SD and studied downstream pathways. We found that acute i.c.v. injections of NMU into the hypothalamus negatively regulate food intake and body weight and increase core body temperature in F344 rats, consistent with findings in nonseasonal mammals. Furthermore, the central administration of NMU into F344 rats held in SD has a direct effect on *Dio2* mRNA expression, mimicking the effects of TSH but, in addition, NMU also regulates Wnt/β-catenin signalling in the hypothalamus.

## Materials and methods

### Animals and ethics statement

Male rats aged 4–5 weeks were obtained from Harlan Sprague–Dawley Inc. (Indianapolis, IN, USA). For the surgical experiment, they were exposed to SD (8:16 h light/dark cycle) immediately after arrival and, for the photoperiod experiment, they were exposed to a 12:12 h light/dark cycle after arrival. Rats were kept in groups of four in constant temperature (21 ± 2 °C) until surgery or photoperiod switch. Food [CRM (P) Rat and Mouse Breeder and Grower, standard pelleted diet (Special Diet Services, Witham, UK)] and water were provided *ad lib*. All experimental procedures were performed under strict adherence to UK home office regulations and in accordance with the Animals (Scientific Procedures) Act, 1986. The animal experiments were licensed and approved by the UK Home Office and approved by the local ethical review committee.

### Surgical procedure

At 10–11 weeks of age (236 ± 20 g), rats were randomly divided into weight-matched groups, anaesthetised by inhalation (2.5–3.0% isoflurane) and placed in a Kopf stereotaxic frame (David Kopf, New York, NY, USA). For i.c.v. cannulation, permanent 22-gauge stainless steel guide cannulae (Plastics One Inc., Roanoke, VA, USA) were implanted into the third ventricle of the hypothalamus (0.8 mm posterior to the bregma line on the mid-sagittal line, 6.5 mm below the outer surface of the scull). Stereotaxic coordinates were calculated using the rat brain atlas of Paxinos and Watson 14. Three stainless steel screws (AgnTho's, Lindingoe, Sweden) were inserted into the cranium and the cannula was fixed to these with dental cement (AgnTho's). After surgery, the animals received 5 ml of 0.9% saline for circulatory support and 1 mg/kg Metacam (Boehringer Ingelheim Vetmedicia GmbH, Ingelheim, Germany) and 150 mg/kg Synolox (Pfizer Limited, Tadworth, UK) and were singly housed. Before surgery and after postoperative recovery, rats were habituated to the injection process by daily handling sessions. Rats were injected under conscious conditions after careful handling to avoid any stressful influence. For i.c.v. injections, compounds were injected in a volume of 5 μl over 1 min using a 28-gauge stainless steel injector (Plastics One) placed in and projecting 1 mm below the tip of the cannula. Cannula placement was confirmed by a positive dipsogenic response to 100 ng of angiotensin II (Sigma-Aldrich, Poole, UK) in 5 μl of saline 1 week after surgery. Only animals with confirmed third ventricle cannula placement were included in the data analysis.

### Central NMU administration experiment

One week after the angiotensin II test, NMU (5.0 nmol/5 μl saline; Bachem, Saffron Walden, UK; n = 4) or vehicle (5 μl saline; n = 5) was administered i.c.v. on three consecutive days at Zeitgeber time (ZT) 3 (i.e. 3 h after lights on). Immediately after i.c.v. injections, animals were presented with a pre-weighed amount of chow. Food consumption was measured every 24 h and body weight was determined before the first injection and 24 h post injection. Core body temperature was monitored remotely in undisturbed animals using i-button® devices (Maxim Integrated Products, San Jose, CA, USA) that were implanted into the peritoneum at the same time as i.c.v. cannulation. Core temperature was measured every 30 min throughout the experiment. Rats were killed 24 h after the last NMU injection at ZT3 by decapitation following isoflurane inhalation. Guide cannulae were carefully removed and brains were immediately dissected, frozen on dry ice and stored at −80 °C.

### TSH infusion experiment

During i.c.v. cannulation, micro-osmotic pumps (Alzet, Durect Corporation, Cupertino, CA, USA; model 1002, flow rate 0.25 μl/h) were implanted s.c. and connected to the i.c.v. cannula. Bovine TSH (1 mIU/day; Sigma-Aldrich; n = 5) or vehicle (saline; n = 4) was infused over a 14-day period. Food intake was measured daily and body weight was measured 3× weekly. Rats were killed after 14 days of TSH infusion at ZT3 by decapitation following isoflurane inhalation. Cannula placement was confirmed with dye administration (5 μl Bromophenol blue; Sigma-Aldrich) at the end of the experiment. Only rats with confirmed third ventricle cannula placement were included in the data analysis. Testes were dissected and paired testes weight was recorded.

### Photoperiod experiment

In the photoperiod experiment, rats aged 7–8 weeks were randomly divided into two groups of four and transferred to either SD or LD (16:8 h light/dark cycle) photoperiod rooms 2 weeks after acclimatisation. In the rooms, the original lights-on time stayed the same; thus, photoperiods were changed by shortening or lengthening the duration before the lights-off times. After 28 days in photoperiod, single-housed rats were anaesthetised using isoflurane inhalation and killed by decapitation at ZT3. Hypothalami were immediately removed on wet ice, frozen on dry ice and stored at −80°C until use.

### *In situ* hybridisation

mRNA levels of *Dio2*, *Dio3*, *Dickkopf 3 (Dkk3)* and *secreted frizzled-related protein 2 (sFrp2)* were analysed in coronal sections of the hypothalamus of F344 rats. These were cut and *in situ* hybridisation was performed using ^35^S-labelled riboprobes as previously described in detail 15. Riboprobe templates were prepared as explained previously for the genes *Dio2* and *Dio3* 16 and *Dkk3* and *sFrp2* 10. Briefly, ^35^S-labelled antisense riboprobes were generated from 100–150 ng of amplified inserts by T7 or T3 RNA polymerase as appropriate. ^35^S-labelled sense riboprobes were synthesised from complementary DNA template strands; no signal was detected using sense riboprobes. Slides were apposed to Biomax MR film (Kodak, Rochester, NY, USA) for 3–7 days, as appropriate. Autoradiographic films were scanned at 1200 d.p.i. and analysed using image-pro plus, version 7.0 (Media Cybernics UK, Marlow, UK) using integrated optical density of the signal relative to a ^14^C microscale (Amersham, Pharmacia Biotech UK Ltd, Little Chalfont, UK). Relative mRNA levels were calculated by defining SD mRNA abundance values as 1.

### RT^2^ PCR Profiler™ polymerase chain reaction (PCR) array

Total RNA was isolated from 60–80 mg of hypothalamic tissue of rats from the photoperiod experiment, using an RNeasy Mini Kit (Qiagen, Valencia, CA, USA) with on-column DNase treatment (Qiagen) in accordance with the manufacturer's specification. The yield and purity of the RNA was quantified with a NanoDrop ND-1000 spectrophotometer (Thermo Scientific, Wilmington, DE, USA) and a Bioanalyzer 2011 (Agilent Technologies, Santa Clara, CA, USA). Equal amounts of total RNA (1 μg) were copied into cDNA using the RT^2^ First Strand kit (Qiagen). Quantitative reverse transcription-PCR was performed using the RT^2^ SYBR Green ROX™ qPCR Mastermix (Qiagen) for use with the 7500 Fast Real Time PCR System (Applied Biosystems, Foster City, CA, USA). The reaction mix was prepared in accordance with the manufacturer's instructions and added to each well of the RT^2^ Profiler™ Rat WNT Signaling Pathway PCR Array (PARN-043; SABiosciences, Frederick, MD, USA). The reaction conditions were initial denaturation for 10 min at 95 °C, followed by 40 cycles of denaturation at 95 °C for 15 s and annealing at 60 °C for 1 min. Relative gene expressions were calculated using rt^2^ profiler pcr data analysis, version 3.4 (http://pcrdataanalysis.sabiosciences.com/pcr/arrayanalysis.php) using five endogenous control genes (*Actb, B2m, Hprt1, Ldha* and *Rplp1*) as internal controls. Fold change values are presented as the mean fold change for genes in LD relative to SD photoperiod samples.

### Statistical analysis

Data were analysed by a two-tailed Student's t-test or two-way factorial anova (treatment group × time interaction) with Tukey's honestly significant difference post-hoc test as appropriate using sigmaplot, version 12.0 (Systat Software Inc., Chicago, USA). Grubb's test was used to test for potential outliers. One data value was identified as an outlier (P = 0.00163) and removed from the analysis. The results are presented as the mean ± SEM. P < 0.05 was considered statistically significant.

## Results

### Effect of TSH on thyroid hormone signalling and physiology

**Figure 1:**
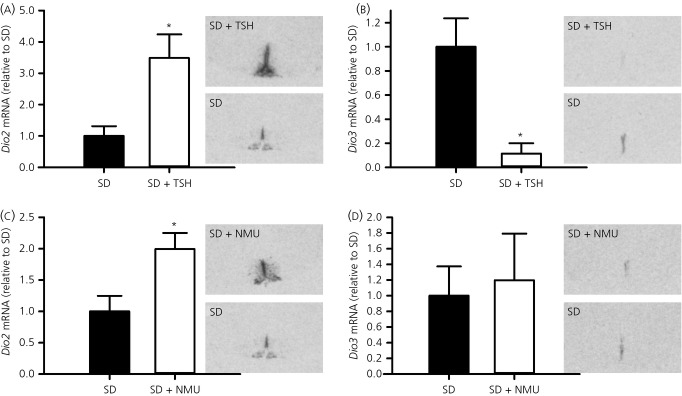
Regulation of hypothalamic deiodinase gene expression by thyroid-stimulating hormone (TSH) and neuromedin U (NMU). (a) *Dio2* and (b) *Dio3* mRNA expression in the ependymal region around the third ventricle in F344 rats after 14 days of TSH (1.0 mIU/day; n = 5) or vehicle (saline; n = 4) infusion. (c) *Dio2* and (d) *Dio3* mRNA expression in the ependymal region around the third ventricle in F344 rats after three consecutive days of NMU (5.0 nmol; n = 4) or vehicle (saline; n = 5) injections at ZT3. F344 rats were acclimated in short days (SD) (8:16 h light/dark cycle) before TSH or NMU administration. Densitometric analyses (bar charts) and representative autoradiographic images from *in situ* hybridisation are shown for each gene. Data shown are the mean ± SEM. *P < 0.05. ZT, Zeitgeber time.

To confirm that TSH regulates *Dio2* mRNA expression in the ependymal region around the third ventricle in F344 rats similar to sheep and mice 3,4, we examined the effects on *Dio2* mRNA expression after 2 weeks of TSH infusion (1.0 mIU/day) into the third ventricle of the hypothalamus of SD housed F344 rats. Using *in situ* hybridisation, we showed that *Dio2* mRNA expression was increased by 3.5-fold in the ependymal layer after 2 weeks of TSH administration (t-test, P = 0.028) (Fig.1a). Second, we investigated *Dio3* mRNA expression in the ependymal layer and found that *Dio3* mRNA expression was also strongly controlled by TSH, with its expression being down-regulated in TSH relative to saline-treated F344 rats (t-test, P = 0.012) (Fig.1b).

**Figure 2:**
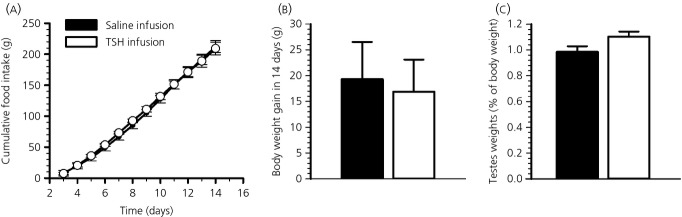
Effects of thyroid-stimulating hormone (TSH) on food intake, body weight and testes weights. *Ad lib.* fed F344 rats held in short days (SD) (8:16 h light/dark cycle) received an i.c.v. infusion of saline [n = 4; (●) or (■)] or 1.0 mIU/day TSH [n = 5; (○) or (□)] for 14 days via osmotic minipumps (flow rate 0.25 μl/h). (a) Food intake was measured daily from 3 days after surgery. (b) Body weight gain calculated as the difference between body weights measured on the day of minipump implantation and 14 days later. (c) Paired testes weights are shown as a percentage of body weight. Data shown are the mean ± SEM.

We also investigated whether 2 weeks of TSH infusion were sufficient to drive physiological changes in body and testes weights and food intake. Following 2 weeks of exposure to TSH (1.0 mIU/day), food intake did not differ between TSH-infused and saline-infused F344 rats (t-test, P = 0.83) (Fig.2a). TSH treatment also had no effect on body weight (t-test, P = 0.81) (Fig.2b). However, a tendency towards an increase in paired testes weight was observed in TSH infused F344 rats, although this failed to reach statistical significance (t-test, P = 0.094) (Fig.2c).

### Effect of NMU on thyroid hormone signalling

To evaluate whether NMU has the potential to regulate TH deiodinase gene expression in the ependymal region around the third ventricle in F344 rats, we examined *Dio2* and *Dio3* mRNA expression after 3 days of i.c.v. injections of NMU (5.0 nmol) into SD housed F344 rats. Relative to saline-injected F344 rats, *Dio2* gene expression in the ependymal region was almost doubled by NMU treatment (P = 0.028) (Fig.1c). *Dio3* mRNA expression was highly variable between animals and was not significantly affected (P = 0.777) (Fig.1d).

### Physiological response to NMU administration

**Figure 3:**
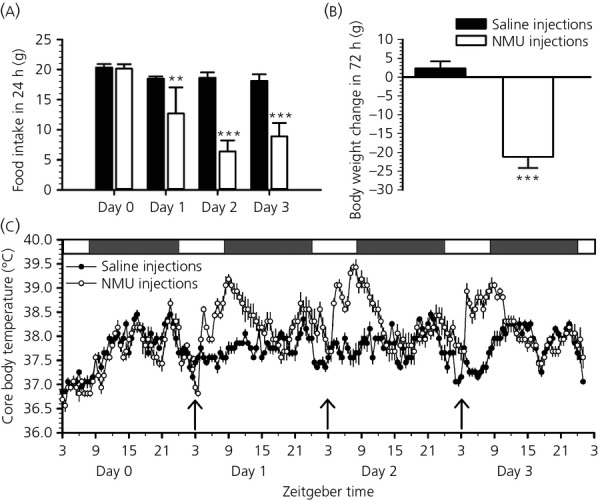
Effects of neuromedin U (NMU) on food intake, body weight and core body temperature. *Ad lib*. fed F344 rats held in short days (SD) (8:16 h light/dark cycle) received an i.c.v. injection of saline [n = 5; (■) or (●)] or 5.0 nmol of NMU [n = 4; (□) or (○)] at ZT3 on 3 consecutive days. (a) Food intake was measured every 24 h starting the day before injections (day 0). (b) Body weight gain calculated as the difference between body weights measured before the first injection and 72 h later. (c) Core body temperature was measured every 30 min throughout the experiment with i.p. implanted i-buttons. Arrows indicate the time of i.c.v. injections of saline or NMU. The white and grey bars at the top of the graph indicate the light and dark phases. Data shown are the mean ± SEM. **P < 0.01, ***P < 0.001. ZT, Zeitgeber time.

The physiological response to NMU was investigated in photoperiod-sensitive F344 rats held in SD. Three days of i.c.v. injections of NMU into satiated F344 rats caused a marked reduction in food intakes. Food intakes decreased compared to saline-injected control rats almost immediately, whereas, after 3 days, intakes were decreased to only half of control intakes (two-way anova, treatment group × time interaction P < 0.001) (Fig.3a). These changes in food intakes were accompanied by a significant decrease in body weight over 3 days of NMU administration (t-test, P < 0.001) (Fig.3b). Core body temperature significantly increased within 1 h after each NMU injection and remained elevated by approximately 1–2 °C for 7–8 h, before dropping to levels of saline injected rats (two-way anova, treatment group × time interaction, P *<* 0.001) (Fig.3c).

### Wnt/β-catenin signalling genes

Recently, we showed that two members of the Wnt/β-catenin pathway are under strong photoperiodic control in the hypothalamus of F344 rats 10. To investigate this further, we used Rat WNT Signaling Pathway RT^2^ profiler PCR arrays to investigate whether other Wnt/β-catenin signalling genes are significantly changed by photoperiod. Significant changes in gene expression were defined using the selection criteria of more than two-fold change, P < 0.05 (Student's t-test) and CT values of < 30. Based on these criteria, detectable PCR products were obtained for 78/84 genes and, from these, we found that eight Wnt signalling genes were significantly up-regulated by LD photoperiod (Table1).

**Table 1:** Up-regulated Wnt/*β*-Catenin Signalling Genes in Long-Day (LD) Compared to Short-Day (SD) Photoperiod.

Gene name	Gene symbol	Fold change*	P value†
*Wingless-type MMTV integration site family member 9b*	Wnt9b	6.85	0.0465
*Secreted frizzled-related protein 2*	Sfrp2	3.89	0.0471
*Dishevelled, dsh 3*	Dvl3	3.24	0.0013
*Dickkopf 3*	Dkk3	2.91	0.0399
*Lymphoid enhancer binding factor 1*	Lef1	2.57	0.0332
*Frizzled homolog 9*	Fzd9	2.36	0.0141
*Transcription factor 3*	Tcf3	2.19	0.0367
*Wingless-type MMTV integration site family member 9a*	Wnt9a	2.12	0.0430

* Mean fold change (>2.00) values for genes in LD relative to SD photoperiod samples (n = 4 per photoperiod group).

† P value: two-tailed Student's t-test, P < 0.05.

### Effect of NMU and TSH on Wnt/β-catenin signalling genes

**Figure 4:**
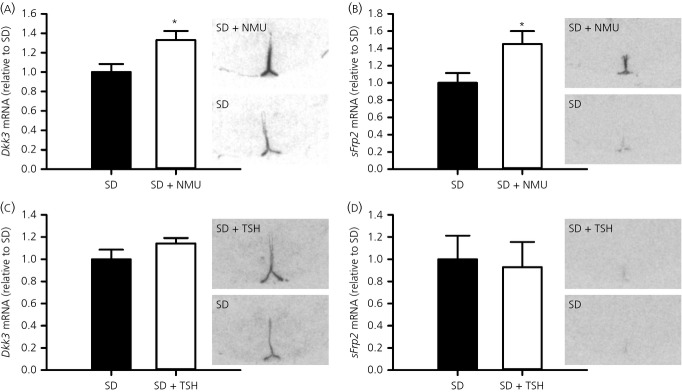
Regulation of Wnt/β-catenin signalling genes by neuromedin U (NMU). (a) *Dkk3* and (b) *sFrp2* mRNA expression in F344 rats after three consecutive days of NMU (5.0 nmol; n = 4) or vehicle (saline; n = 5) injections at ZT3. (c) *Dkk3* and (d) *sFrp2* mRNA expression in the ependymal region around the third ventricle in F344 rats after 14 days of TSH (1.0 mIU/day; n = 5) or vehicle (saline; n = 4) infusion. F344 rats were acclimated in short days (SD) (8:16 h light/dark cycle) before NMU or TSH administration. Densitometric analyses (bar charts) and representative autoradiographic images from *in situ* hybridisation are shown for each gene. Data shown are the mean ± SEM. *P < 0.05. ZT, Zeitgeber time.

Next, we explored whether NMU was involved in the regulation of Wnt/β-catenin pathway genes. Using *in situ* hybridisation, we found that *Dickkopf 3* (*Dkk3)* mRNA expression was significantly increased in NMU-treated compared to saline-treated F344 rats (t-test, P = 0.033) (Fig.4a). The same pattern was evident with regards to *secreted frizzled-related protein 2* (*sFrp2)*. Compared to saline-treated F344 rats, *sFrp2* mRNA was up-regulated after NMU treatment (t-test, P = 0.044) (Fig.4b).

Neither *Dkk3*, nor *sFrp2* showed a difference after TSH treatment (t-test, P = 0.176 and 0.824, respectively) (Fig.4c,d).

## Discussion

In the last decade, accumulating data from a variety of seasonal species have helped to unravel the primary effects of photoperiod on the neuroendocrine hypothalamus. It is now generally accepted that, in mammals, photoperiod, through melatonin, regulates TSH production in the pars tuberalis of the pituitary and, in turn, TSH regulates hypothalamic TH availability, which is considered to be the basis of long-term seasonal changes in energy metabolism 9.

In the present study, we provide further new evidence in support of this general mechanism in photoperiod-sensitive F344 rats. F344 rats, exposed to SD photoperiod, which would be expected to have low hypothalamic TSH availability and result in low Dio2 expression, received osmotic minipumps releasing TSH for 2 weeks, thereby mimicking LD conditions. We found that, after 2 weeks of TSH infusion, *Dio2* mRNA was significantly up-regulated in the ependymal layer of the third ventricle, consistent with previous studies 2–4. To date, a direct effect of TSH administration has only been reported for *Dio2*, although it has been suggested that TSH production in the pars tuberalis might also control the expression of *Dio3* in the tanycytes 3. In the present study, we have also extended our understanding of the role of TSH in the regulation of hypothalamic TH metabolism, by showing for the first time that *Dio3* expression was negatively regulated by TSH in F344 rats. In SD housed F344 rats, where *Dio3* levels are high, 2 weeks of TSH infusion reduced *Dio3* to levels, as would be expected for LD housed rats. Although the regulation of TH deiodinases in the hypothalamus by photoperiod appears to be a conserved feature, the relative importance of Dio2 and Dio3 appears dependent upon the seasonal species. In Syrian hamsters, transfer from SD to LD induces Dio2 expression but Dio3 expression has not been observed in either photoperiod 5,16. In Siberian hamsters, on the other hand, both Dio2 and Dio3 change in response to photoperiod, although the changes for Dio3 are more pronounced 16–18. In the common vole, the deiodinase enzymes are not expressed at the same stage of the seasonal cycle, resulting in a situation where Dio3 dominates in SD, whereas Dio2 dominates in LD conditions 8. In both the European hamster and the F344 rat, a switch from SD to LD results in an increase in Dio2 expression, which is correlated with a decrease in Dio3 expression 6,7. In the short-lived seasonal rodents discussed above, reports suggest Dio2/3 expression appears to be limited to the ependymal region 9. By contrast, in the long-lived, short-day breeding Soay sheep, the pattern of gene expression distribution changes throughout the hypothalamus with the seasons and notably, at certain times, an increase in Dio2 and decrease in Dio3 occurs without any obvious change in TSH 19. It was concluded that, in the sheep, the pars tuberalis through TSH, may regulate only the summer to autumn transition and not the winter to spring one. Our results show that, in F344 rats, pars tuberalis-derived TSH triggers a reciprocal regulation of both deiodinase enzymes in the tanycytes of the hypothalamus. The basis of this reciprocal regulation is at present unclear because the signal transduction mechanisms leading to the inhibition of Dio3 by TSH are unknown. It is known that TSH activates Dio2 gene expression through Gs-protein/cyclic AMP-dependent signalling via the TSH-R 2. Although TSH-R has been shown to also couple to Gq proteins 20, the relationship of this or alternative pathways, to the regulation of Dio3 expression remains to be established.

In the present study, the effects of TSH on Dio2 and Dio3 were measured at 2 weeks because, in F344 rats, this time frame is sufficient to result in a significant difference in body weight and food intake between SD and LD photoperiods 15,21. Despite significant reciprocal changes in *Dio2* and *Dio3* expression at this time point, no effect was observed on either food intake or body weight following 2 weeks of chronic TSH infusion. This outcome could either reflect that TSH alone cannot fully mimic the photoperiodic response in F344 rats or that the duration of the TSH infusion was too short to result in a change in these parameters. Although photoperiodic changes in Dio2 and Dio3 in the ependymal layer have been reported to be very rapid 5,7,21, the effect on downstream physiological changes can take up to several weeks in many seasonal animals 5,16,22. It is also possible that the invasive nature of the surgery has masked a physiological effect. The weight gain of the TSH and saline-infused rats was lower than would be expected in nonsurgical rats in SD after 14 days 15. Paired testes weights also did not differ significantly after 2 weeks of central TSH administration, although there appeared to be a trend towards higher testes weights in TSH-administered F344 rats. This may suggest that a significant effect may be seen after a longer period of infusion. This would be consistent with the findings in quail, where i.c.v. injections of TSH induces gonadal recrudescence 2.

In the present study, we hypothesised that the neuropeptide NMU might also be involved in the photoperiodic response because, first, the NMU gene is expressed in the pars tuberalis and its receptor NMU_2_ is expressed in the ependymal layer of the third ventricle 10,13,23, which is very similar to the expression profile of TSH and its receptor 7. Indeed, Aizawa *et al.* 24 have recently shown that NMU is expressed in the same cells in the PT as TSH. Second, similar to TSH and its receptor, NMU and NMU_2_ are under strong photoperiodic control in F344 rats 10. Yet, in contrast to these similarities, NMU is known for its role in the negative regulation of body weight and food intake 11. To clarify the role of NMU in photoperiodic animals, we injected NMU into the third ventricle of the hypothalamus of F344 rats and analysed downstream pathways.

As a first step, we examined the physiological response to 3 days of NMU injections into the third ventricle of the hypothalamus of F344 rats. Three days were considered appropriate in the present study because the acute effects of NMU administration on food intake and body weight are well documented over this period, yet, at the same time, robust changes in NMU mRNA expression in the pars tuberalis, as well as gene expression changes associated with the photoperiodic response (e.g. Dio2), also occur in this period 7. Consistent with the findings obtained in previous studies conducted in nonphotoperiodic rats and mice, we found a pronounced reduction in body weight and food intake after 3 days of NMU administration 11. Furthermore, NMU injections acutely increased core body temperature in F344 rats, as previously reported in nonphotoperiodic rats and mice 12,23,25,26, indicating an increase in energy expenditure. Although these findings are consistent with the known anorexigenic characteristics of NMU in the brain of nonphotoperiodic animals, they are apparently at odds with the high levels of NMU expression observed in the pars tuberalis in LD, which are associated with high levels of food intake and body weight in photoperiod-sensitive F344 rats 10. To explore whether NMU can act as a mediator of photoperiod, we next investigated whether NMU has the potential to regulate hypothalamic Dio2 expression because Dio2 converts the inactive form of thyroid hormone T4 into the bioactive form T3 and T3 availability appears to underlie changes in seasonal physiology 9. In the present study, we found a significant increase in *Dio2* expression in the ependymal layer of the third ventricle after 3 days of NMU injections but no effect on *Dio3* expression. Given the established functional relationship between the pars tuberalis and the tanycytes around the third ventricle for TSH 27, it is equally feasible that NMU could pass from the pars tuberalis to the third ventricle where it can also contribute to the photoperiodic regulation of *Dio2* expression in the ependymal layer. The lack of effect on *Dio3* expression relative to the effect of TSH could reflect the difference in time-points chosen for the measurements (i.e. 3 days versus 14 days) or be a genuine difference in action of the two peptides.

The apparently disparate responses to NMU delivered i.c.v., in terms of inhibitory effects on food intake and body weight relative to the stimulatory effects on *Dio2* expression, which underpins long-term body weight increase in photoperiodic rats, has a potentially simple explanation. In the rat, there are two major areas of NMU_2_ receptor expression in the hypothalamus. The first is in the paraventricular nucleus (PVN) and the other is in the cells lining the third ventricle 12,13,28. NMU is synthesised in the arcuate nucleus, and from there, fibres project to the PVN 29 and, in response to i.c.v. injection of NMU, Fos-like immunoreactivity can be detected in the PVN 30. Furthermore, when NMU is delivered directly into the PVN, this triggers acute suppression of food intake, amongst a variety of other behavioural responses 31,32. This suggests that NMU_2_ receptors in the PVN have a role in food intake and body weight responses to NMU, where under natural conditions, NMU would be synthesised in the ARC and delivered to the PVN through neural projections.

The NMU_2_ receptors expressed in the ependymal cells in the hypothalamus are likely to mediate the changes in *Dio2* expression in response to NMU seen in this region, with the source of the NMU activating these receptors coming from the pars tuberalis of the pituitary. Evidence suggests that this novel pars tuberalis–ependymal endocrine axis function is unlikely to be involved in acute feeding responses. This is because NMU gene expression in the pars tuberalis is increased in fasted Sprague–Dawley rats, when leptin levels should be low and is down-regulated after central leptin administration 33. These responses drive NMU expression in the pars tuberalis in the wrong direction if it is to provide a compensatory food intake drive following energy or leptin deficit.

Another factor in support of the role of NMU in the pars tuberalis-ependymal axis for photoperiodic responses is its effects on Wnt/β-catenin signalling. Previously, we have shown that two members of the Wnt/β-catenin signalling pathway are under strong photoperiodic control in the ependymal layer of F344 rats 7,10. In the present study, we extended this analysis to show that eight genes from the Wnt pathway were significantly up-regulated in LD compared to SD conditions. These genes included *sFrp2* and *Dkk3*, both of which are expressed in the ependymal region and arcuate nucleus of the hypothalamus of F344 rats 10. Besides Wnt9a and Wnt9b, all other genes from the Wnt pathway identified are members of the β-catenin pathway (canonical Wnt pathway). The biological significance of canonical Wnt signalling in the hypothalamus is currently unknown but, in a seasonal context, it is tempting to speculate that this pathway, together with the retinoic acid system 10, might be involved in cellular and structural remodelling of the adult hypothalamus. This is because canonical Wnt/β-catenin signalling has been strongly implicated in synaptic maintenance and function, as well as adult neurogenesis 34. There is now clear evidence for neurogenesis in the adult hypothalamus 35–37 and our own studies have shown that cell proliferation is regulated by photoperiod in F344 rats 38. Such changes would be more consistent with the longer-term physiological changes characteristic of seasonal species than with the more rapid changes associated with acute energy balance. However, it remains to be established whether and how the Wnt pathway is involved in seasonal remodelling of the hypothalamus.

The importance of the Wnt pathway in the present study is that we show that both *Dkk3* and *sFrp2*, secreted glycoproteins that regulate the canonical Wnt/β-catenin signalling, are both up-regulated after the central administration of NMU. This suggests that NMU from the pars tuberalis is not only important for the regulation of hypothalamic Dio2 expression, but also has an important function with respect to regulating Wnt/β-catenin signalling within the hypothalamic ependymal/tanycyte cells. It is noteworthy that neither Dkk3, nor sFrp2 expression was altered by TSH administered i.c.v.. This may reflect the differences in the time-points (3 days versus 14 days) when assessing the responses between NMU and TSH or it may be a difference in the function of the two peptides. Because differences in the expression of these genes tend to increase in direct proportion to the time in photoperiod 7, the latter may offer the more likely explanation.

Thus, in conclusion, the data from the present study support the view that the two main sites of NMU_2_ expression in the rat hypothalamus may serve distinct functions. Namely NMU_2_ receptors in the PVN are involved in acute feeding and other behavioural responses, whereas those in the ependymal layer are involved in long-term physiological responses typified in photoperiodic F344 rats. Intracerebroventricular administration is therefore a blunt instrument to deliver NMU for the study of its physiological effects, and the neuroanatomy and source of NMU, as well as the location of its receptors, must be taken into account when interpreting its effects.

Overall, we provide evidence that both TSH and NMU, derived from the pars tuberalis, serve important roles in the photoperiodic responses of F344 rats. Because these responses are mainly long-term changes in energy balance, growth and reproduction, these data shed new light on the neuroendocrine regulatory mechanisms that are distinct from the more acute mechanisms normally studied. Because both TSH and NMU are expressed in the pars tuberalis and Dio2 is expressed in the ependymal/tanycycte cells of the hypothalamus of nonseasonal rats, these findings may have important implications for long-term neuroendocrine regulation in nonphotoperiodic species.
